# Red cell distribution width to albumin ratio and mortality in acute pulmonary thromboembolism

**DOI:** 10.17305/bb.2024.10791

**Published:** 2024-12-06

**Authors:** Berrin Zinnet Eraslan, Sümeyye Kodalak Cengiz, Özlem Saniye İçmeli, Seda Beyhan Sagmen, Sevda Şener Cömert

**Affiliations:** 1 Department of Chest Diseases, University of Health Sciences, Kartal Dr. Lütfi Kırdar City Hospital, İstanbul, Türkiye

**Keywords:** Pulmonary embolism, red cell distribution width to albumin ratio, RAR, red cell distribution width, RDW, prognosis, mortality

## Abstract

The red cell distribution width (RDW)/albumin ratio (RAR) is an inflammation-based prognostic biomarker. To date, its prognostic value in patients with pulmonary thromboembolism (PTE) has been investigated in only one study. This study aimed to assess the effect of RAR on mortality in patients with PTE. Patients admitted for PTE between 2017 and 2023 were retrospectively reviewed. The data collected included demographic information, comorbidities, clinical findings, RDW, albumin, troponin, D-dimer levels, and in-hospital and 30-day mortality outcomes. RAR was calculated by dividing the RDW by albumin. A total of 190 patients were included in the study, of whom 83 (43.7%) were male. The mean age was 63 years (range: 23–89), and the mean RAR was 4.48% ± 1.68% /g/dL. A positive correlation was observed between RAR and both age and troponin level, whereas inverse correlations were noted with systolic blood pressure (sBP), diastolic blood pressure (dBP), and oxygen saturation (SpO2). Using a cutoff value of 5.294 determined by ROC analysis, patients with RAR ≥ 5.294 had a significantly shorter mean survival time than those with RAR value < 5.294 (16.310 months vs 35.163 months; log-rank test, *P* < 0.001). Multivariate Cox regression analysis identified malignancy (hazard ratio [HR], 4.213; 95% confidence interval [CI], 1.103–16.090, *P* ═ 0.035) and RAR (HR: 1.295, 95% CI: 1.035–1.621, *P* ═ 0.024) as independent predictors of survival. In conclusion, an RAR value ≥ 5.294 was associated with significantly shorter survival, underscoring its potential utility as a prognostic marker in clinical practice for non-massive PTE.

## Introduction

Acute pulmonary thromboembolism (PTE) is the third most common cause of death from cardiovascular diseases, following heart attack and stroke [1]. Right ventricular dysfunction resulting from impaired filling and/or decreased right ventricular outflow, triggered by acute vascular overload after PTE, is the leading cause of mortality in severe PTE [2].

Prognostic factors commonly used to assess hemodynamically stable acute symptomatic PTE patients include the pulmonary embolism severity index (PESI) and simplified PESI (sPESI), calculated using clinical parameters [3]. Given the complexity of the original PESI, which includes 11 differently weighted variables, a simplified version known as sPESI was developed and validated [4]. Similar to the original PESI, the strength of sPESI lies in its ability to reliably identify patients at low risk of 30-day mortality. The prognostic performance of sPESI has been confirmed in observational cohort studies [5]. An sPESI score of 0 was considered low-risk, with a 30-day mortality risk of approximately 1.0%. In contrast, an sPESI score ≥ 1 is classified as a high-risk group, where the 30-day mortality risk increases to 10.9% [6].

In addition, in patients with PE, markers of right ventricular dysfunction, such as transthoracic echocardiography, brain natriuretic peptide (BNP), NT-proBNP, and troponin (a marker of myocardial damage), are commonly used for prognostic evaluation [7–9].

The red cell distribution (RDW) to albumin ratio (RAR) is a novel inflammatory biomarker that has been associated with mortality in diseases such as chronic obstructive pulmonary disease, myocardial infarction, diabetic ketoacidosis, and acute respiratory distress syndrome [10–13].

However, to date, only one study has investigated the association between RAR and PTE [14]. Given the limited evidence in the literature, this study aimed to further explore the prognostic value of RAR in PTE by evaluating its effect on mortality.

Through this analysis, we aimed to determine whether RAR could serve as a valuable prognostic marker in clinical practice.

## Materials and methods

### Study design and setting

**Figure 1.**
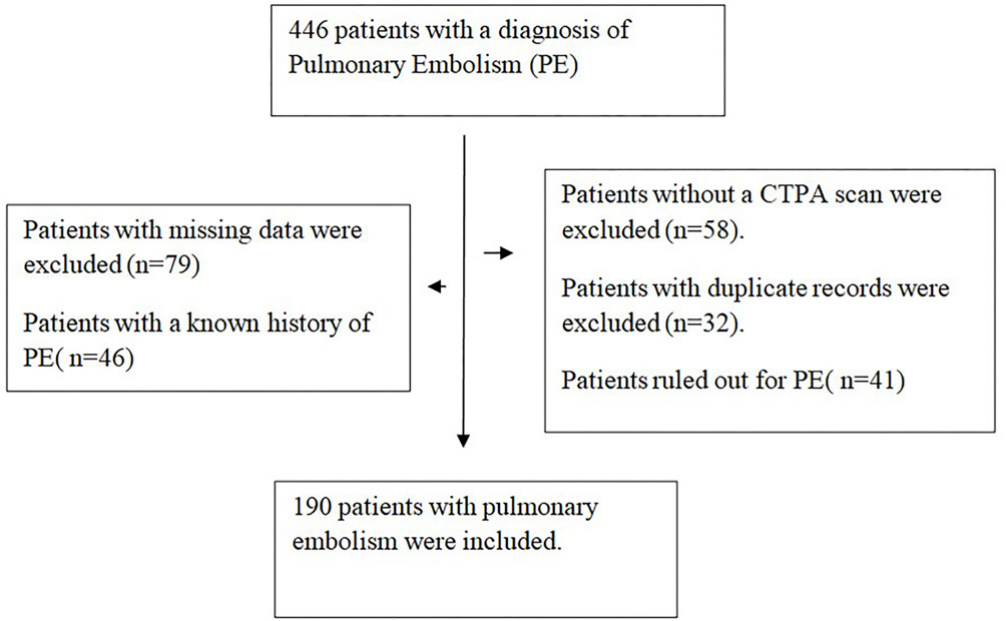
**Flowchart diagram.** Abbreviation: CTPA: Computed tomography pulmonary angiography.

In this retrospective study conducted at a tertiary care hospital in Turkey, patients hospitalized with a diagnosis of pulmonary embolism in our hospital between 2017 and 2023 were reviewed retrospectively. Patients with massive pulmonary embolism and those with an indication for thrombolytic therapy were not included in the study because they were monitored in the intensive care unit by a separate medical team. Consequently, it was not possible to ensure standardized data collection for these patients. Therefore, the focus of this study was on non-massive PTE cases managed in our general ward to maintain data consistency and enhance the reliability of the analysis. Additionally, patients with incomplete data or uncertain embolism diagnosis were excluded from the study. Patients aged ≥ 18 years with PE confirmed by computed tomography pulmonary angiography (CTPA) were included in this study (Figure 1). Patient data collected from the medical records included gender, age, length of hospital stay (days), systolic blood pressure (sBP), diastolic blood pressure (dBP), heart rate, oxygen saturation (SpO2), serum creatinine (Scr), D-dimer levels, red cell distribution width (RDW), albumin levels, troponin levels, comorbidities (hypertension, diabetes mellitus [DM], chronic cardiovascular disease, chronic kidney disease, etc.), malignancy, atrial fibrillation (AF), history of surgery within the last month, deep vein thrombosis (DVT), fracture, and immobility. All blood samples for RDW, albumin, troponin, and D-dimer were collected within the first 24 h of hospital admission to ensure consistency in the timing of the measurements. D-dimer levels were analyzed using a turbidimetric method on a fully automated Sysmex CS-2500 device (Sysmex Corporation, Norderstedt, Germany). CBCs were measured using an LH750 hematology analyzer (Beckman Coulter, Brea, CA, USA). Troponin levels were analyzed using a Beckman Coulter DXI-800 immunoassay analyzer (Beckman Coulter, Brea, CA, USA). Albumin was analyzed using a Beckman Coulter AU 5800 analyzer (Beckman Coulter, Brea, CA, USA) following the manufacturer’s instructions.

Additionally, in-hospital mortality, simplified PESI (sPESI), mortality from the time of diagnosis, and patients’ last seen dates were recorded. Finally, RAR was calculated by dividing the RDW (%) by albumin (g/dL).

The sPESI was calculated based on six clinical variables: age > 80 years, history of cancer, chronic cardiopulmonary disease, heart rate ≥ 110 beats per minute, sBP less than 100 mmHg, and arterial oxygen saturation (SpO2) < 90%. Each of these factors was assigned 1 point, and the total score was used to classify patients into two risk categories: low-risk (sPESI ═ 0) and high-risk (sPESI ≥ 1). This scoring system was used to stratify the 30-day mortality risk in patients with PTE [1].

In patients with PTE, the primary outcomes were in-hospital mortality and 30-day all-cause mortality, including mortality specifically related to PTE. These outcomes were selected to evaluate the prognostic value of RAR in predicting short-term and in-hospital mortality. The secondary outcomes included the association of RAR with overall survival, assessed using Kaplan–Meier survival analysis. The specific cutoff value for RAR was determined by ROC curve analysis and was later used to evaluate its impact on survival outcomes.

### Ethical statement

The research protocol was approved by the ethics board of the University of Health Sciences Kartal Dr. Lütfi Kırdar City Hospital and adhered to the principles outlined in the Declaration of Helsinki (Approval Date: 29/05/2023; Approval Number: 2023/514/250/20). Given the retrospective nature of this study, the requirement for written informed consent was waived.

### Statistical analysis

The data obtained in this study were analyzed using the SPSS (Statistical Package for Social Sciences) version 25.0 software package. Descriptive analyses are presented as frequency data using numbers (*n*) and percentages (%), while numerical data are shown as mean ± standard deviation, median (minimum-maximum), and confidence interval (95% CI). The normality of the distribution of numerical data was assessed using the sample size and the Kolmogorov–Smirnov tests. The distribution of numerical data that did not conform to a normal distribution in the two independent groups was analyzed using the Mann–Whitney *U* test. The comparison of numerical data that did not conform to a normal distribution in more than two independent groups was analyzed using the Kruskal–Wallis test. For post-hoc analysis of the Kruskal–Wallis test results, which were found to be significant, the Mann–Whitney *U* test was used with– the Dunn–Bonferroni correction. The relationship between the two numerical variables was analyzed using Spearman’s correlation analysis. The cutoff point determination properties of the variables were evaluated using receiver operating characteristic (ROC) curve analysis. Kaplan–Meier analysis was performed to determine the effect of variables on patient survival time. Multivariate Cox regression analysis was used to determine the effect of the independent prognostic factors on patient survival. The results were evaluated at a 95% CI, with significance considered at *P* < 0.05.

**Table 1:** Demographic and clinical characteristics of patients with pulmonary embolism

**Variables**		***n***	**%**
Sex, *n* (%)	Male	83	(43.68)
	Female	107	(56.32)
		**Mean ± SD**	**Median (Min–Max)**
Age (years)		62.2 ± 16.65	63 (23–89)
Length of hospital stay (days)		8.83 ± 5.18	8 (2–39)
Systolic blood pressure (mmHg)		119.03 ± 22.82	111 (80–229)
Diastolic blood pressure (mmHg)		72.35 ± 14.58	70 (50–140)
Heart rate (bpm)		87.11 ± 16.65	84 (53–140)
Oxygen saturation (SpO2) %		92.64 ± 5.5	94 (64–100)
Serum creatinine mg/dL		0.9 ± 0.41	0.83 (0.26–4.4)
D-dimer µg/L		7074.83 ± 7898.89	3885 (160–35,000)
Red cell distribution width (%)		15.05 ± 3.33	14.4 (3.56–30.2)
Albumin g/dL		3.48 ± 0.67	3.5 (1–4.8)
RAR*		4.48 ± 1.68	4.12 (1.23–14.72)
Troponin (ng/mL)		0.07 ± 0.22	0.02 (0–2.06)
		**n**	**%**
Comorbid disease ≥1		118	62.11
Diabetes mellitus		42	24.4
Hypertension		73	42.4
Cardiovascular disease		22	11.6
Cerebrovascular disease		12	6.3
Chronic obstructive pulmonary disease		16	8.4
Chronic kidney disease		13	6.84
Malignancy		64	33.68
Atrial fibrillation		18	9.47
history of surgery		24	12.63
Deep vein thrombosis		72	38.10
Fracture		12	6.32
Immobility		74	38.95
In-hospital mortality		12	6.32
sPESI ≥ 1		108	56.84
Mortality (1-month)		22	11.58

Abbreviation: *RAR: Red cell distribution width to albumin ratio.

### G*power sample size calculation

The sample size was calculated using the G*Power program based on an independent samples *t*-test analysis of the distribution of RAR in patients with and without mortality. Assuming an allocation ratio of 1:5 between the two groups (mortality/non-mortality), as in the study by Qiu et al. [10], with a 5% significance level (α), 80% power (1-β), and a medium effect size (0.55), the required sample size was determined to be a minimum of 190 participants, with 32 in Group 1 (mortality) and 158 in Group 2 (non-mortality).

## Results

The study included 190 patients aged between 23 and 89 years, and 83 were men (43.7%). Of these patients, 118 (62.1%) had comorbidities, 64 (33.7%) had cancer, 72 (38.1%) had DVT confirmed using Doppler ultrasound, and 108 (56.8%) had an sPESI score of ≥1. The mean length of hospital stay was 8.83 ± 5.18 days. There were 12 (6.3%) in-hospital deaths, and 22 (11.6%) patients died within 30 days (Table 1).

Comparisons of RDW, albumin, and RAR yielded significant results. Females had higher RDW values than males (*P* ═ 0.021). Patients with comorbidities had higher RDW values (*P* ═ 0.025) and lower albumin values (*P* ═ 0.016). Patients with cancer had higher RDW and RAR values (*P* < 0.001 and *P* < 0.001, respectively) and lower albumin values compared to patients without cancer (*P* < 0.001). Albumin levels were lower in patients with immobility, AF, and DVT compared to those without (*P* < 0.001, *P* ═ 0.034, and *P* ═ 0.018, respectively) (Table 2).

**Figure 2.**
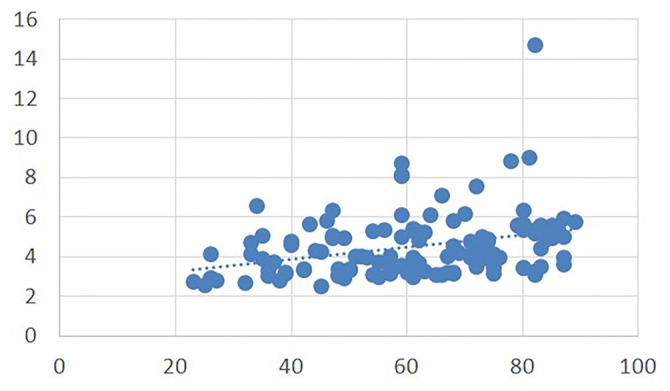
**Correlation graph between RAR and age.** Scatter plot showing that RAR was significantly and positively correlated with age (*P* < 0.001). Abbreviation: RAR: Red cell distribution width to albumin ratio.

Patients who died in the hospital had higher RDW and RAR values (*P* ═ 0.037 and *P* ═ 0.031, respectively) and lower albumin values (*P* ═ 0.006). Patients with a simple PESI ≥ 1 had higher RDW and RAR values and lower albumin values. Analysis of 30-day mortality showed that non-survivors had significantly higher RAR (*P* ═ 0.005) and lower albumin levels (*P* < 0.001) (Table 2).

The correlations of RDW, albumin, and RAR with age, length of hospital stay, sBP and dBP, heart rate, SpO2%, Scr, D-dimer, and troponin are shown in Table 3.

**Table 2:** Comparisons among RDW, albumin, and RAR and other variables

		**RDW**		***P***	**Albumin**		***P***	**RAR**		***P***
		**Mean ± SD**	**Median (Min–Max)**		**Mean ± SD**	**Median (Min–Max)**		**Mean ± SD**	**Median (Min–Max)**
Gender	Male	14.7 ± 3.18	13.7 (11.3–30.2)	**0.021**	3.46 ± 0.65	3.55 (1.7–4.6)	0.743	4.46 ± 1.54	4.03 (2.54–9)	0.712
	Female	15.32 ± 3.44	14.7 (3.56–26.5)		3.5 ± 0.69	3.5 (1–4.8)		4.49 ± 1.79	4.14 (1.23–14.72)	
Comorbidity ≥1	No	14.89 ± 3.45	13.3 (11.5–30.2)	**0.025**	3.62 ± 0.7	3.7 (1–4.6)	**0.016**	4.26 ± 1.44	4 (2.54–9)	0.114
	Yes	15.15 ± 3.26	14.65 (3.56–26.5)		3.41 ± 0.64	3.4 (1.7–4.8)		4.61 ± 1.8	4.2 (1.23–14.72)	
Malignancy	No	14.32 ± 2.99	13.7 (3.56–26.5)	**<0.001**	3.61 ± 0.62	3.65 (1.8–4.8)	**<0.001**	4.14 ± 1.66	3.75 (1.23–14.72)	**<0.001**
	Yes	16.74 ± 3.49	15.9 (13–30.2)		3.25 ± 0.69	3.15 (1–4.6)		5.21 ± 1.48	5.03 (3.23–9)	
Atrial fibrillation	No	15.02 ± 3.44	14.15 (3.56–30.2)	0.222	3.51 ± 0.68	3.6 (1–4.8)	**0.034**	4.46 ± 1.73	4.03 (1.23–14.72)	0.187
	Yes	15.27 ± 1.75	15.1 (13.3–20.1)		3.22 ± 0.49	3.2 (2.5–4.3)		4.71 ± 0.83	4.86 (3.51–5.96)	
History of surgery	No	14.97 ± 3.44	14.2 (3.56–30.2)	0.155	3.48 ± 0.65	3.5 (1.7–4.8)	0.451	4.51 ± 1.76	4.12 (1.23–14.72)	0.726
	Yes	15.63 ± 2.26	15.3 (13–20.3)		3.51 ± 0.82	3.75 (1–4.5)		4.2 ± 0.9	4.03 (2.98–5.75)	
Deep vein thrombosis	No	15.01 ± 2.66	14.5 (11.3–26.5)	0.878	3.55 ± 0.69	3.6 (1–4.8)	**0.018**	4.4 ± 1.67	4.05 (2.54–14.72)	0.140
	Yes	15.11 ± 4.55	14.2 (3.56–30.2)		3.32 ± 0.62	3.3 (1.8–4.4)		4.75 ± 1.77	4.23 (1.23–9)	
	unknown	14.71 ± 2.9	14 (11.5–20.6)		3.79 ± 0.49	3.85 (2.9–4.4)		4.02 ± 1.39	3.45 (2.91–7.1)	
Fracture	No	15.2 ± 3.22	14.6 (3.99–30.2)	0.065	3.48 ± 0.68	3.5 (1–4.8)	0.691	4.53 ± 1.69	4.14 (1.33–14.72)	0.111
	Yes	12.28 ± 4.2	13.5 (3.56–17.1)		3.57 ± 0.43	3.5 (2.9–4.2)		3.49 ± 1.23	3.75 (1.23–5.18)	
Immobility	No	15.07 ± 3.07	13.9 (11.3–30.2)	0.278	3.62 ± 0.69	3.7 (1–4.8)	**<0.001**	4.29 ± 1.37	3.97 (2.54–8.72)	0.051
	Yes	15.01 ± 3.84	14.65 (3.56–26.5)		3.27 ± 0.58	3.3 (1.7–4.5)		4.85 ± 2.15	4.74 (1.23–14.72)	
Chronic kidney disease	No	15 ± 3.36	14.2 (3.56–30.2)	0.274	3.49 ± 0.68	3.5 (1–4.8)	0.384	4.45 ± 1.67	4.09 (1.23–14.72)	0.386
	Yes	15.84 ± 2.77	14.7 (13.5–21.6)		3.34 ± 0.6	3.4 (2.2–4.2)		5.01 ± 1.86	4.45 (3.62–9)	
In-hospital mortality	No	14.96 ± 3.34	14.2 (3.56–30.2)	**0.037**	3.52 ± 0.64	3.6 (1.7–4.8)	**0.006**	4.42 ± 1.66	4.03 (1.23–14.72)	**0.031**
	Yes	16.93 ± 2.71	16 (14.2–21.6)		2.9 ± 0.81	3 (1–4.3)		5.72 ± 1.81	5.53 (3.53–9)	
sPESI ≥ 1	No	14.32 ± 2.32	13.6 (11.3–21.1)	**0.001**	3.75 ± 0.64	3.8 (1.8–4.8)	**<0.001**	3.97 ± 1.2	3.51 (2.54–8.72)	**<0.001**
	Yes	15.72 ± 3.94	15.1 (3.56–30.2)		3.29 ± 0.62	3.3 (1–4.7)		4.91 ± 1.91	4.76 (1.23–14.72)	
Mortality (1-month)	No	14.93 ± 3.06	14.1 (3.99–30.2)	0.055	3.56 ± 0.62	3.6 (1.8–4.8)	**<0.001**	4.28 ± 1.27	4.03 (1.33–8.72)	**0.005**
	Yes	16.14 ± 5.26	15.2 (3.56–26.5)		2.87 ± 0.76	2.9 (1–4.3)		6.16 ± 3.31	5.59 (1.23–14.72)	

Abbreviations: RDW: Red cell distribution width; RAR: Red cell distribution width to albumin ratio.

**Table 3:** Correlations of RDW, albumin, and RAR with other variables

**Variables**		**RDW**	**Albumin**	**RAR**
Age (years)	r	0.236	−0.394	0.342
	p	**0.006**	**<0.001**	**<0.001**
Length of hospital stay (days)	r	−0.002	−0.135	0.083
	p	0.985	0.070	0.353
Systolic blood pressure (mmHg)	r	−0.240	0.126	−0.225
	p	**0.006**	0.092	**0.011**
Diastolic blood pressure (mmHg)	r	−0.60	0.141	−0.194
	p	0.066	0.059	**0.030**
Heart rate (bpm)	r	0.025	−0.237	0.163
	p	0.778	**0.002**	0.071
Oxygen saturation (SpO2) %	r	−0.166	0.296	−0.196
	p	0.056	**<0.001**	**0.027**
Serum creatinine (mg/dL)	r	0.032	−0.007	0.023
	p	0.713	0.927	0.801
D-dimer (µg/L)	r	−0.120	−0.163	0.011
	p	0.296	0.090	0.928
Troponin (ng/mL)	r	0.313	−0.342	0.408
	p	**0.001**	**<0.001**	**<0.001**

Abbreviations: RDW: Red cell distribution width; RAR: Red cell distribution width to albumin ratio.

**Table 4:** ROC analysis of RDW, RAR, and albumin parameters

	**RDW**	**RAR**	**Albumin**
**Cut-off value**	≥15.05	≥5.294	≤3.25
**AUC (95% CI)**	0.716 (0.592–0.839)	0.808 (0.679–0.938)	0.768 (0.655–0.881)
**Sensitivity (95% CI)**	84.62 (54.55–98.08)	76.92 (46.19–94.96)	80.95 (58.09–94.55)
**Specificity (95% CI)**	64.41 (55.07–73.00)	82.14 (73.78–88.74)	70.81 (63.13–77.70)
**PPV (95% CI)**	20.75 (15.77–26.81)	33.33 (23.33–45.10)	26.56 (20.84–33.20)
**NPV (95% CI)**	97.44 (91.34–99.28)	96.84 (91.89–98.81)	96.61 (92.15–98.58)
**Accuracy (95% CI)**	66.41 (57.64–74.42)	81.60 (73.68–87.96)	71.98 (64.86–78.37)
**Youden index**	49.03	59.06	51.76

Abbreviations: CI: Confidence interval; AUC: Area under the curve; PPV: Positive predictive value; NPV: Negative predictive value; ROC: Receiver operating characteristic; RDW: Red cell distribution width; RAR: Red cell distribution width to albumin ratio.

**Figure 3.**
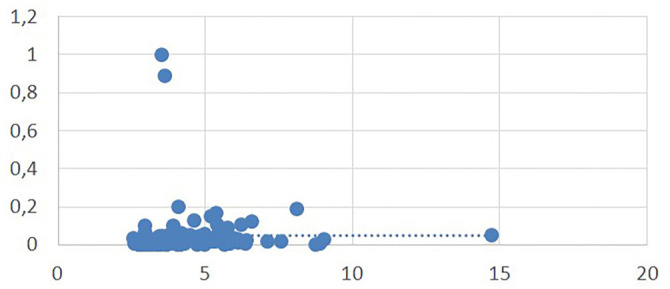
**Correlation graph between RAR and troponin.** Scatter plot showing that RAR was significantly and positively correlated with troponin (*P* < 0.001). Abbreviation: RAR: Red cell distribution width to albumin ratio.

RAR was positively correlated with age (*P* < 0.001) and troponin level (*P* < 0.001) (Figures 2 and 3). Inversely correlated with sBP and dBP and SpO2%. There was no correlation between RAR and the length of hospital stay (*P* ═ 0.353, *r* ═ 0.083).

ROC curve analyses were conducted to assess the predictive value of RDW, RAR, and Albumin levels in predicting mortality in PTE.

**Figure 4.**
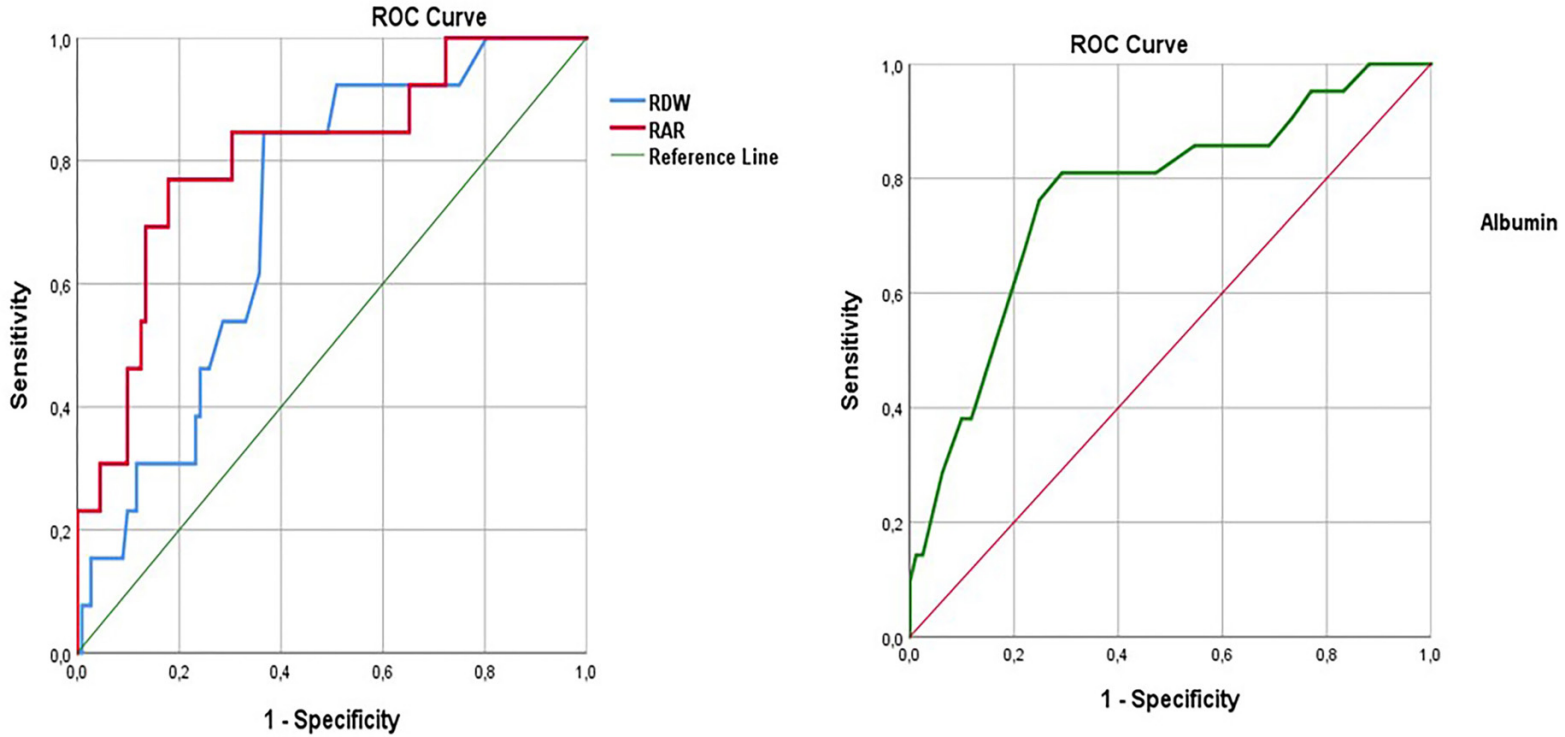
**ROC Curves of RDW, RAR, and albumin for predicting mortality.** RDW demonstrated a moderate predictive ability (AUC ═ 0.716), whereas both RAR (AUC ═ 0.808) and albumin (AUC ═ 0.768) showed strong predictive performance. Abbreviations: ROC: Receiver operating characteristic; RDW: Red cell distribution width; RAR: Red cell distribution width to albumin ratio.

ROC curve analysis for RDW yielded an AUC of 0.716 (95% CI: 0.592–0.839), indicating a moderate predictive value for mortality. The optimal cut-off value was determined to be ≥15.05, with a sensitivity of 84.62% (95% CI: 54.55–98.08) and a specificity of 64.41% (95% CI: 55.07–73.00). This suggests that RDW is a useful marker for predicting mortality, particularly with high sensitivity, although its specificity was moderate (Figure 4 and Table 4).

**Figure 5.**
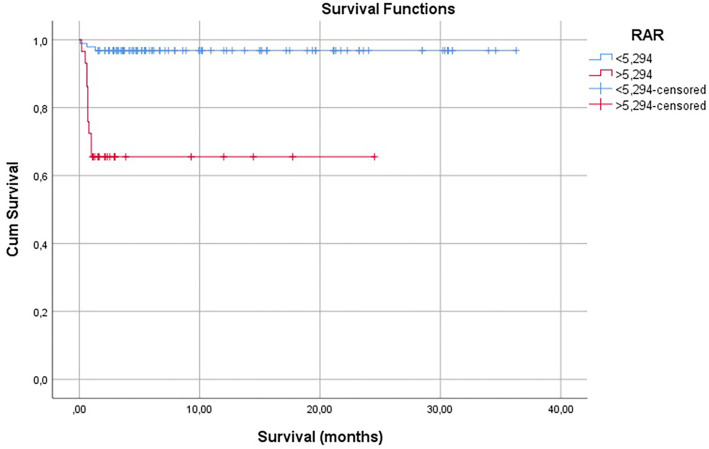
**Kaplan–Meier analysis of the impact of RAR 5.294 cut-off value on survival.** The mean survival time was 35.163 months (95% CI: 33.897--36.429) for patients with RAR < 5.294 compared to 16.310 months (95% CI: 12.185--20.436) for those with RAR ≥ 5.294. The difference in survival was statistically significant (log-rank test, *P* < 0.001). Abbreviation: RAR: Red cell distribution width to albumin ratio.

For RAR, the ROC analysis revealed an AUC of 0.808 (95% CI: 0.679–0.938), demonstrating a strong predictive value. The cut-off value for RAR was established at ≥5.294, yielding a sensitivity of 76.92% (95% CI: 46.19–94.96) and a specificity of 82.14% (95% CI: 73.78–88.74). The high specificity and balanced sensitivity suggest that RAR is a reliable predictor of mortality in this patient population (Figure 4 and Table 4).

Finally, the ROC curve for albumin levels showed an AUC of 0.768 (95% CI: 0.655–0.881), indicating a strong predictive value for mortality. The optimal cut-off for albumin was identified as ≤3.25, with a sensitivity of 80.95% (95% CI: 58.09–94.55) and a specificity of 70.81% (95% CI: 63.13–77.70). This suggests that lower albumin levels are associated with an increased risk of mortality, highlighting their potential as prognostic markers (Figure 4 and Table 4).

Kaplan–Meier survival analysis was performed to evaluate the impact of RAR on the overall survival of patients with PTE. The survival curves demonstrated a significant difference in survival outcomes between patients with RAR ≥5.294 and those with RAR <5.294, which was determined as the optimal cut-off value through ROC analysis.

Patients with RAR ≥5.294 had notably lower survival rates than those with RAR <5.294. The survival curve for the higher RAR group declined sharply within the first few months, and then plateaued, indicating a higher risk of early mortality. In contrast, the survival curve for the lower RAR group remained relatively stable, reflecting a better overall survival rate.

The mean survival time was 35.163 months (95% CI: 33.897–36.429) for patients with RAR <5.294, compared to 16.310 months (95% CI: 12.185–20.436) for those with RAR ≥5.294. The effect of the RAR cutoff value of 5.294 on survival was found to be statistically significant (log-rank test, *P* < 0.001) (Figure 5).

All demographic, comorbidity, and laboratory parameters of the patients were analyzed using univariate Cox regression analysis. The variables that were significant in the univariate Cox regression analysis, along with the results of the multivariate Cox regression analysis conducted using these variables, are presented in Table 5. According to the results of the univariate Cox regression analysis, the presence of DM (OR: 2.469), malignancy (OR: 3.060), immobility (OR: 2.367), increased heart rate (OR: 1.038), and increased simple PESI score (OR: 5.239) were identified as factors that significantly increased mortality and affected survival. According to the results of multivariate Cox regression analysis, the presence of malignancy and an increase in RAR were identified as factors that significantly increased mortality and affected survival (*P* ═ 0.005).

**Table 5:** Cox regression analysis of independent prognostic factors on survival

**Survival time**				
	**Univariate cox-regression**		**Multivariate cox-regression**	
	**OR (95% CI)**	***P***	**OR (95% CI)**	***P***
Diabetes mellitus	2.469 (1.055–5.776)	**0.037**	0.464 (0.088–2.447)	0.366
Malignancy	3.060 (1.308–7.162)	**0.010**	4.213 (1.103–16.090)	**0.035**
İmmobility	2.376 (1.015–5.560)	**0.046**	2.154 (0.654–7.099)	0.207
Heart rate	1.038 (1.016–1.061)	**0.001**	1.032 (0.998–1.066)	0.067
sPESI	5.239 (1.550–17.708)	**0.008**	1.504 (0.278–8.128)	0.636
RAR	4.660 (2.009–10.806)	**<0.001**	1.295 (1.035–1.621)	**0.024**

Abbreviation: RAR: Red cell distribution width to albumin ratio.

## Discussion

This study aimed to determine whether RAR, a novel inflammatory biomarker, can predict mortality in patients with acute PTE. The results showed that the RAR was a strong independent predictor of mortality**.** These results underscore the potential of RAR as a valuable biomarker for risk stratification in PTE management.

The objective of our study was to investigate the prognostic value of RAR in patients with PTE who were initially hospitalized in a general ward. Our findings demonstrate that RAR is a strong predictor of mortality in this subset of non-ICU patients with PTE and non-massive pulmonary embolism. This aligns with the results reported by Ding et al. [14], who focused on patients in the ICU. However, by excluding severe cases, such as those with massive PTE requiring thrombolytic therapy, the generalizability of our study was limited to non-massive PTE cases managed outside the ICU.

The sPESI is a widely used scoring system designed to stratify the risk of mortality in patients diagnosed with PTE [1]. In our study, we found that sPESI was a significant predictor of mortality in univariate analysis, with an odds ratio of 5.239 (95% CI: 1.550–17.708, *P* ═ 0.008), making it the variable with the highest impact on mortality among the significant factors. Additionally, both RAR and malignancy were identified as significant predictors of mortality, further emphasizing their role in risk stratification. However, in the multivariate analysis, the association between sPESI and mortality was not statistically significant (OR: 1.504; 95% CI: 0.278–8.128, *P* ═ 0.636). These findings highlight the importance of comprehensive risk assessment in managing PTE, where sPESI can serve as a starting point but should be considered alongside other clinical variables.

RDW is a parameter of complete blood count and represents a noninvasive and cost-effective test. Elevated RDW levels are associated with anemia, hematological disorders, oxidative stress, and general erythropoiesis disorders [15]. Additionally, high RDW levels have been found to correlate with C-reactive protein (CRP) and erythrocyte sedimentation rate (ESR), suggesting that RDW may be considered a marker of inflammation [16]. Furthermore, anisocytosis, which is a consequence of elevated RDW, may impair capillary blood flow [17]. This disruption of capillary flow can hinder proper blood circulation and increase the propensity for thrombosis. Previous studies have reported RDW to be correlated with mortality in patients with acute PTE [18, 19]. A meta-analysis by Xing et al. [20] showed that mortality was higher in the group with higher RDW levels.

Albumin, which is synthesized in the liver, performs numerous essential functions. It serves as a marker of nutritional status and inflammation while also regulating oncotic pressure in the circulatory system. Additionally, it possesses antioxidant properties, neutralizes free radicals, and prevents cellular damage [21–24]. Consequently, hypoalbuminemia may represent a hyperinflammatory state that contributes to the increased incidence of venous thromboembolism. Recent studies have demonstrated that hypoalbuminemia is not only associated with an increased risk of VTE but also serves as an independent predictor of mortality in patients with acute PTE [25–27].

In this study, RAR, calculated by dividing RDW by albumin, was found to be a more effective predictor of mortality in patients compared to RDW or albumin alone. This conclusion is supported by our ROC analysis, in which RAR demonstrated a higher AUC value and a greater Youden index, indicating its superior prognostic value.

Cancer is a well-known risk factor for PTE, and the presence of malignancy significantly increases the risk of mortality in PTE patients [28, 29]. Additionally, cancer is a significant risk factor for all-cause mortality following a venous thromboembolism [30]. In our study, we found that malignancy was an independent predictor of mortality in patients with PTE. Univariate Cox regression analysis identified malignancy as a factor that nearly tripled the risk of mortality (OR: 3.060; 95% CI: 1.308–7.162, *P* ═ 0.010). This association persisted in the multivariate analysis, in which malignancy was shown to increase the risk of mortality by approximately four times (OR: 4.213; 95% CI: 1.103–16.090, *P* ═ 0.035). These findings underscore the adverse impact of cancer on overall survival in patients with PTE and highlight the importance of incorporating malignancy into risk assessment models for these patients.

In our study, heart rate was found to be a significant predictor of mortality in univariate analysis (OR: 1.038; *P* ═ 0.001), but not in the multivariate model when other variables such as RAR and malignancy were included (OR: 1.032; *P* ═ 0.067). This suggests that heart rate by itself may be associated with mortality risk but does not have sufficient power as an independent predictor when important factors such as RAR and malignancy are included. RAR and malignancy, which remained significant in the multivariate model, may have a stronger or more direct association with mortality. Heart rate may be partially influenced by these conditions or indirectly contributes to mortality through pathways that overlap with these conditions.

In our study, the RDW values were significantly higher in females than in males (*P* ═ 0.021). This finding may be attributed to several biological factors that are specific to female physiology. Hormonal differences, particularly the effects of estrogen, can influence erythropoiesis and inflammatory responses, potentially leading to increased RDW. Additionally, the higher prevalence of iron deficiency in females may contribute to greater variability in red blood cell size (anisocytosis), which could further explain the observed differences.

Our study had several limitations. First, it was a single-center retrospective study, which may have introduced a selection bias, potentially affecting the accuracy and generalizability of our findings. To enhance the robustness of these results, they should be confirmed in multicenter prospective studies with a larger and more diverse patient population. Second, all data were based on single measurements, limiting our ability to assess the changes over time. Additionally, the absence of echocardiographic (ECHO) and BNP data restricted our ability to identify patients with PTE with accompanying right ventricular dysfunction. This limitation hindered our ability to differentiate high-risk submassive PTE cases related to right ventricular strain. As a result, the potential for some high-risk patients to be overlooked may limit the completeness of our prognostic evaluation. Furthermore, our initial power analysis estimated the need for 32 mortality cases to achieve adequate statistical power. However, only 22 cases were observed in our cohort. This discrepancy may be due to the exclusion of patients with massive pulmonary embolism, who typically have a higher mortality risk.

## Conclusion

In this study, RAR was identified as an independent prognostic marker for mortality in patients with acute PTE with a significant impact on survival outcomes. The presence of malignancy and increased RAR were the most significant factors associated with decreased survival, as demonstrated using multivariate Cox regression analysis. These findings suggest that RAR along with malignancy should be considered in risk stratification and management of patients with PTE.

## Data Availability

The datasets supporting the conclusions of this article and its supporting information are available from the corresponding author upon reasonable request.
